# Using Tablet Computers to Increase Patient Engagement With Electronic Personal Health Records: Protocol For a Prospective, Randomized Interventional Study

**DOI:** 10.2196/resprot.4672

**Published:** 2016-09-06

**Authors:** S Ryan Greysen, Yimdriuska Magan Mendoza, Jaime Rosenthal, Ronald Jacolbia, Alvin Rajkomar, Herman Lee, Andrew Auerbach

**Affiliations:** ^1^ University of California, San Francisco Division of Hospital Medicine San Francisco, CA United States; ^2^ University of California, San Francisco School of Nursing San Francisco, CA United States; ^3^ Midwestern University Glendale, AZ United States

**Keywords:** mobile health, patient engagement, older adults

## Abstract

**Background:**

Inadequate patient engagement in care is a major barrier to successful transitions from the inpatient setting and can lead to preventable adverse events after discharge, particularly for older adults. While older adults may be less familiar with mobile devices and applications, they may benefit from focused bedside training to engage them in using their Personal Health Record (PHR). Mobile technologies such as tablet computers can be used in the hospital to help bridge this gap in experience by teaching older, hospitalized patients to actively manage their medication list through their PHR during hospitalization and continue to use their PHR for other post-discharge tasks such as scheduling follow-up appointments, viewing test results, and communicating with providers. Bridging this gap is especially important for older, hospitalized adults as they are at higher risk than younger populations for low engagement in transitions of care and poor outcomes such as readmission. Greater understanding of the advantages and limitations of mobile devices for older adults may be important for improving transitions of care.

**Objective:**

To better understand the effective use of mobile technologies to improve transitions in care for hospitalized, older adults and leverage these technologies to improve inpatient and postdischarge care for older adults.

**Methods:**

We will compare an intervention group with tablet-based training to engage effectively with their PHR to a control group also receiving tablets and basic access to their PHR but no additional training on how to engage with their PHR.

**Results:**

Patient enrollment is ongoing.

**Conclusions:**

Through this grant, we will further develop our preliminary dataset and practical experience with these mobile technologies to catalyze patient engagement during hospitalization.

**ClinicalTrial:**

ClinicalTrials.gov NCT02109601; https://clinicaltrials.gov/ct2/show/NCT02109601 (Archived by WebCite at http://www.webcitation.org/6jpXjkwM8)

## Introduction

### Engagement in Hospital Care and Personal Health Records

Low engagement in discharge planning and poor understanding of discharge medications are major barriers to promoting successful transitions of care for older adults at many US hospitals. As many as 1 in 5 older patients experience an adverse event after discharge, many of which are likely preventable [1-3]. The overall impact of these poor transitions on costs and outcomes of care is also substantial: 15% to 30% of Medicare patients experience unplanned readmissions with related cost exceeding $17 billion annually [4]. Accordingly, a new Medicare policy to reduce readmission rates was introduced as a core cost-saving component of the Affordable Care Act in 2012, and many hospitals have initiated intense efforts to improve transitions of care [5,6]. Many hospitals have deployed discharge coordinators and extended discharge training for hospital staff to improve transitions, but uses of mobile technologies to add or extend the value of these human interventions have been understudied [7,8].

Many existing technology-enabled interventions have relied heavily on provider uses of the electronic medical record (EMR) to increase the completion and accuracy of key transition tasks such as scheduling appointments, communicating with providers, and completing medication reconciliation [9-11]. While EMR-based interventions have been successful in reducing preventable adverse drug events from inaccurate medication reconciliation, they are clinician-centered and do not actively engage patients. This is a missed opportunity not only to enhance patients’ understanding of their medication list but also to learn about using their personal health record (PHR) to continue actively monitoring their medication list after discharge. Furthermore, interventions focused only on medication reconciliation do not address other important aspects of care transitions such as managing follow-up appointments, viewing test results, and communicating with providers. An ideal patient-centered mobile health intervention would facilitate patients’ ability to view and interact with their medication list as well as empower patients to engage in these other aspects of postdischarge care through active use of their PHR.

### Mobile Technology, Hospital Care, and Older Adults

The widespread use of mobile computers (laptops, tablets, and smartphones) in everyday activities has prompted surprisingly few studies of mobile devices in the care of hospitalized patients. Health care provider use of tablet computers to collect clinical registration data, distribute educational materials to patients, or do clinical work (eg, check labs, write notes) has been studied primarily in outpatient settings [12-17]. To date, however, there are very few studies of tablet use by *patients* in the *inpatient* setting, and none have focused on challenges specific to older, hospitalized adults [18]. To address these knowledge gaps, we propose two aims that will create data needed to assess advantages and limitations of these devices and develop a preliminary dataset and practical experience to build and scale innovative approaches to patient engagement during hospitalization.

### Study Aims and Project Impact on Patient Care

We propose a structured, tablet-based intervention targeted to older patients (aged 50 years or more) admitted to the medical service to enhance PHR use in both inpatient and postdischarge settings for specific tasks. All participants will receive study tablets and access to their PHR. Intervention patients will receive additional training related to using their PHR for specific tasks such as medication management, making appointments, viewing test results, and communicating with providers.

#### Aim 1: To Promote Inpatient Engagement With Their PHR During and After Hospitalization.

We will analyze patients’ use of their PHR throughout hospitalization and also for 7 days after discharge to determine if our intervention affects likelihood of PHR use to view medications, check lab results, view appointments, or send a provider message.

#### Aim 2: To Compare Effects of an Inpatient PHR Engagement Intervention to a Virtual Cohort.

We will compare PHR engagement of our study participants to a virtual cohort of patients who were hospitalized but who were already regular users of their PHR. Patients in this virtual cohort will be identified through review of EMR data only and receive no intervention—they will serve as a benchmark for all participants (both intervention and control) in our study.

We hypothesize that those in the intervention group will be more likely than the control group to use their PHR while they are inpatients and after discharge and that patients in both the intervention and control groups will be more likely to use their PHR in these settings than the virtual cohort. In addition to testing these hypotheses, our project is designed to have significant impact on patient engagement with their care during hospitalization at our hospital. We will also add significant value to existing efforts by our health system to increase patient use of existing technologies: our system uses the PHR application called MyChart (Epic Systems Corp), a Web-based patient portal that allows patients to access to their PHR and perform health care tasks such as sending messages to providers, requesting appointments, viewing test results, and reviewing their medications. The overall objective of this research is to study the use of mobile platforms to improve transitions in care for older, hospitalized adults.

## Methods

### Study Design, Participants, and Setting

This is a prospective, randomized interventional study of tablet computers (iPad 16 GB 3^rd^ generation Model A1430) as a new platform to engage older patients in actively using their PHR during and after hospitalization. We focused on tablet computers because they are light and mobile, which is helpful in the inpatient setting (eg, patients laying in gurneys for hours, patients with limited strength). The PHR used at our institution is mobile-friendly and easily accessible via Web browsers commonly used on tablets, desktops, laptops, and smartphones. We plan to enroll 100 hospitalized patients over 12 months on two general medical units at a large, academic teaching hospital (University of California, San Francisco [UCSF] Medical Center). We will also leverage EMR data to compare these 100 patients to a virtual cohort of 400 patients with the same inclusion/exclusion criteria who were already engaged in PHR use; patients in this virtual cohort will have no direct involvement in the study and will serve only as a benchmark for engagement by the 100 patients who actively participate in the study.

Each morning, research assistants will screen for new patients through UCSF’s electronic medical records by searching for medical service. While our study focus is on older patients (aged 50 years or more), we will also enroll patients younger than 50 years in order to compare and contrast patterns of use between these groups. We will include adult, English-speaking, cognitively intact patients on the medical service. To ensure patients selected will be able to use the iPad, we will exclude patients who are reported by the medical team to be cognitively impaired, blind, deaf, or involuntarily hospitalized because of mental illness. We will also exclude those who are unable to understand the study and consent to participation or who are otherwise excluded at the discretion of their medical team.

### Randomization and Intervention

To randomize patients into intervention and control groups, we used an Internet-based coin toss random number generator (Random.org). The research assistant performs this randomization process after patient screening and consent. If the result of the coin toss is heads, the patient is enrolled in the intervention arm; if the result is tails, the patient is enrolled in the control arm. Patients were enrolled Monday through Friday only, no weekends or holidays.

Our project will provide all study participants with tablets and access codes to MyChart while they are hospitalized. Intervention patients will also receive bedside training from study research assistants on key functions of MyChart including how to view medication lists (see Multimedia Appendix 1). Through our preliminary work, we have created a semistructured tutorial for this bedside training that assistants can administer to patients. While this tutorial allows for a basic level of content uniformity for all patients, it is flexible enough to allow assistants to speed up or slow down or otherwise tailor the depth of explanation to the needs of each individual patient (see Multimedia Appendix 2). This study was approved by the UCSF Institutional Review Board and registered at Clinicaltrials.gov [NCT02109601].

### Data Collection and Sources of Data

**Figure 1:**
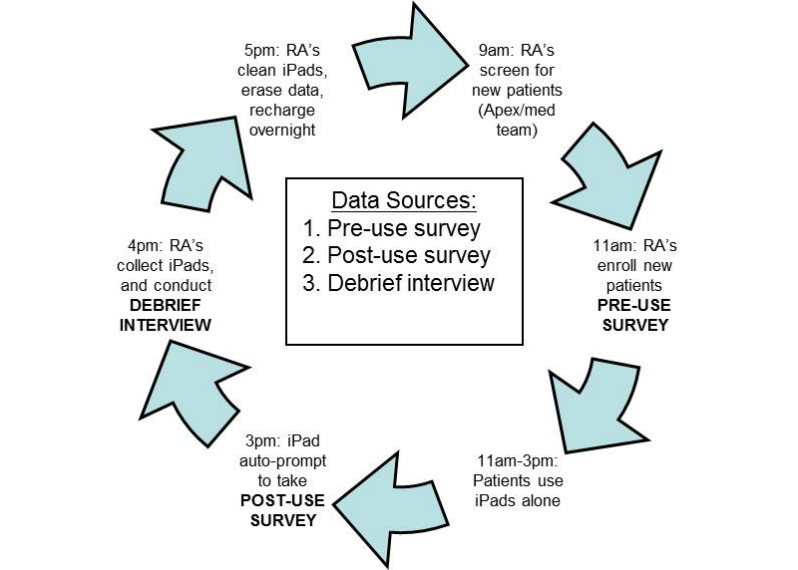
Inpatient iPad tablet daily workflow.

After creating a list of eligible patients, research assistants will approach medical teams during rounds (9:00 AM to 11:00 AM) to ensure that screened patients do not have exclusion criteria (Figure 1). The assistants will then approach patients to explain the study, including basic instructions for tablet use (on/off, opening/closing applications, and typing with touchscreen); intervention patients only will receive additional instruction in the form of the semistructured tutorial. The assistant will then leave the device with the patient and return to recollect it after about 4 to 5 hours. The assistant will perform a debrief interveiw upon return and ask patients to view and verify their preadmission medications using the medication list in their PHR. Success (gross pass/fail) in this task will be defined as patient ability to independently log in, locate their medication list, and verify their prehospitalization prescriptions (MyChart does not show inpatient medications unless they are continued at discharge). We will not require patients to correctly describe indications for every medication to receive credit; they will only be asked to identify which medications are familiar to them and which are not. We will use frequency analysis to compare the percentage of intervention group who are able to complete this task with the control group.

Sources of data will include surveys that will be administered on the device, semistructured debrief interviews administered by assistants, and data on postdischarge access to the PHR from our EMR data repositories. All patients will answer basic questions about demographics, health literacy, and technology use in a preuse survey. The postuse survey will ask about satisfaction with the device to access their PHR and any technical issues. We have developed an interview tool for assistants to use to collect information about patient-reported problems with devices and ask open-ended questions about the overall experience using the tablet. During the debrief interview, the assistant will also ask the patient to demonstrate ability to access their PHR to view and verify their preadmission medications using their PHR medication list. Data about patient use of their PHR is compiled by our EMR in a searchable database (Clarity). We will query Clarity at 7 days to determine access and specific PHR tasks accomplished by all patients both during their hospital stay and for 7 days after discharge. We will also use the Clarity database to identify our virtual cohort of patients who were already engaged with their PHR (1 or more log-ins in the month prior to hospitalization) and were hospitalized during the same 12-month period of our study.

### Data Analysis

Our overall objective is to assess whether inpatients can successfully use iPads for clinically useful tasks such as viewing their medications or labs or messaging their providers by using their PHR. First, we will assess this overall goal using descriptive statistics (frequency analysis) to describe time required to orient patients to tablets and basic use metrics for PHR (time spent using PHR, number of PHR tasks observed by assistant, etc). Next, we will determine the total number of log-ins to the PHR and number of clicks on different domains of the PHR (medications, labs, appointments) by querying the PHR database. We will extract basic information about whether each patient has accessed their PHR (successful log-in), when they first logged in (time from discharge), and how many times they logged in. Additionally, we will extract information about specific tasks accomplished at any point during both inpatient and postdischarge periods. We will use frequency analysis to compare the following for intervention versus control patients: rates of PHR access, time to first log-in, total number of log-ins, and number of specific tasks (refill prescription, make/change appointment, view test result, or send message to provider) accomplished within 7 days. Finally, we will assess patient satisfaction with devices overall and PHR use specifically. We will perform bivariate analyses (chi-square tests) to determine whether ability to perform key PHR tasks (eg, view medications or lab results) vary according to demographics such as age, gender, health literacy, or technology usage (eg, types of devices owned, frequency of Internet use). Although most of our findings will be descriptive, we plan to enroll 100 patients to enable detection of a 10% difference in ability to perform a key inpatient task (medication verification) and one of several key postdischarge PHR tasks (80% power, 2-sided alpha 0.05).

## Results

Preliminary data from our protocol are show in Table 1. To date, we have enrolled 45 patients; approximately half our sample comprises older adults (50-79 years, 22) and about half (24) are female. Most participants reported owning a laptop computer (34, 75%) or smartphone (29, 64%); desktop and tablet computer ownership were lower at 54% (24) and 51% (23), respectively. A strong majority of patients (39, 86%) indicated they access the Internet daily. While 82% (37) reported looking up health information for themselves on the Internet in the past 12 months, only slightly more than half (27, 59%) had used the Internet to communicate with a health care provider and few had used it to schedule a medical appointment (18, 40%) or refill a prescription (17, 37%).

**Table 1:** Participant characteristics (n=45).

		n (%)
Age (years)	
	18-39	9 (19)
	30-49	14 (32)
	50-79	22 (49)
Gender	
	Female	24 (53)
	Male	19 (42)
	Declined/no response	2 (4)
Device ownership	
	Desktop computer	24 (54)
	Laptop computer	34 (75)
	Smartphone	29 (64)
	Tablet computer	23 (51)
Internet use	
	Daily	39 (86)
	Several times a week	3 (6)
	Once a week or less	1 (3)
Prestudy online health tasks	
	Looked up health information	37 (82)
	Communicated with provider	27 (59)
	Scheduled medical appointment	18 (40)
	Refilled prescription	17 (37)
Orientation to iPad	
	Required 15 minutes or less	36 (81)
	Required 16 to 30 minutes	4 (9)
	Required 30 minutes or more	5 (10)
Independently access/navigate PHR on iPad	
	Log in/verify info	28 (62)
	Medications list page	34 (76)
	Medications refills page	18 (40)
	Scheduled appointments page	38 (85)
	Test results page	38 (85)
	Secure messaging page	37 (82)

Among older adults, 70% (15) owned a laptop, 58% (13) owned a desktop computer, 52% (11) owned a smartphone, and 42% (9) owned a tablet. Thus, mobile technologies pose an excellent venue to interact with this patient population. Trained research assistants spent at least 30 minutes with those patients who needed more assistance to become familiar with the tablets, but the majority needed 15 minutes or less, suggesting that most of them felt comfortable with using this technology. Indeed, our findings to date suggest that inpatients of all ages in both intervention and control groups require only minimal orientation from research assistants to use basic (eg, access Internet, check email) as well as more advanced functions (watch videos, complete tasks requiring more advanced navigation): 90% (41) of patients required 30 minutes or less for device orientation and 81% (36) required 15 minutes or less. Additionally, we found that after focused bedside teaching, most patients in our study were able to perform one or more key function in their PHR. These key functions included viewing their medication list (34, 76%), viewing scheduled appointments (38, 85%), viewing test results (38, 85%), and sending a secure message to their primary care provider (37, 82%). Additionally, 75% (34) reported they were satisfied or very satisfied with iPad tablet use for bedside access to their PHR to accomplish these tasks.

Because data is still being collected to compare patient groups within our study (intervention vs control), we compared PHR use among all participants (to date) in both groups to a virtual cohort of patients identified as regular users of their PHR prior to hospitalization. In most areas, both groups were similar, but our study group had significantly higher activity in medication list views (mean 2.12 views vs 1.07) and the virtual cohort had significantly higher use of the provider messaging function (mean 4.34 views vs 1.63) (Table 2).

**Table 2:** Patient characteristics and PHR use in study participants compared to a virtual cohort of regular PHR users.

		Study group (n=45)	Virtual cohort (n=400)	*P* value
Inpatient characteristics				
	Hospital length of stay, days	5.79	4.51	.37
	Previous PHR experience, n (%)	22 (50)	400 (100)	<.01
PHR use during hospitalization				
	Logins per person, mean	2.74	2.99	.64
	Medication tab views, mean	2.12	1.07	<.01
	Test results tab views, mean	1.42	2.21	.28
	Appointments tab views, mean	0.69	1.34	.11
	Provider messaging inbox views, mean	1.63	4.34	.04
PHR use after hospitalization (up to 7 days after discharge)				
	Logins per person, mean	4.04	3.87	.83
	Medication tab views, mean	2.93	1.46	<.01
	Test results tab views, mean	6.28	4.29	.37
	Appointments tab views, mean	2.72	2.01	.32
	Provider messaging inbox views, mean	3.91	6.11	.36

**Table 3:** Adjusted PHR use during hospitalization by study group compared to virtual cohort group.

	Multivariable logistic regression^a^	
	OR for study group vs virtual cohort group mean	95% CI
Medication tab views	2.4	1.5-4.2
Test results tab views	1.1	0.7^-^1.8
Appointments tab views	0.71	0.5^-^1.1
Provider messaging inbox views	0.70	0.5^-^1.1

^a^Adjusted for age, gender, race/ethnicity, length of stay, and prior PHR experience.

In analyses adjusted for confounding, the higher rate of provider messaging seen in Table 3 disappeared with adjustment; however, the higher rate of medication list views persisted (odds ratio [OR] 2.4) (Table 3). In adjusted analyses of PHR use after discharge, there were no significant differences between the virtual cohort and the study group.

## Discussion

### Principal Findings

Our preliminary findings demonstrate relatively high ownership and use of mobile devices among all patients (including those over age 50 years) and relatively low use of these devices for specific health care tasks that can be accomplished via patient PHRs. This gap between high device ownership/use and low engagement in PHR confirms a pattern seen in our previous work and suggests an opportunity to intervene in order to increase PHR engagement during and after hospitalization [19]. Regarding device use, national data from the Pew Research Center show that 55% of all adults own a smartphone and 43% own a tablet; our study shows higher rates, at 64% (29/45) for smartphones and 51% (23/45) for tablets [20]. Although this is only half of our planned total enrollment, this difference in level of tech-savviness may limit the generalizability of our results to less tech-savvy populations if these trends persist in our final sample.

Although we do not yet have data to report on differences between our intervention and control group, we are able to do some preliminary comparisons between both groups (all of whom received a tablet and assistance logging into their PHR) and a virtual cohort of patients who were regular PHR users but received neither tablets nor PHR assistance during hospitalization. Our findings to date show that patients in our study were more likely to use their PHR for viewing the medications during hospitalization. These findings suggest that providing tablets and even basic PHR assistance for patients in the hospital may be an intervention that can improve engagement among inpatients to resemble patterns seen in a group of patients who were already engaged in using their PHR before hospitalization. Once final data collection and analysis are complete for our study, we will explore differences between intervention and control patients and compare each group separately to the virtual cohort as a benchmark for PHR engagement.

### Strengths

While our results are preliminary, we believe this project has potential to be innovative in several ways. First, our study will be one of the first to explore a patient-centered mobile health approach to engage adult patients in hospital and postdischarge settings. Furthermore, by exploring patient experiences with mobile devices, our research will enable us to carry out future projects to engage hospitalized patients in a variety of tasks. For example, we would like to build on experience from the current project to use patient PHRs at bedside in the hospital for more advanced discharge planning in coordination with multidisciplinary team rounds. Finally, our focus on older adults is especially important as they represent a large portion of our health system’s inpatient population, they are at particularly high risk for poor outcomes of transition care, and they are the least likely to be familiar with their PHR or to use it meaningfully without targeted interventions.

### Limitations

Despite these strengths, our study has several limitations which will be important to contextualize our preliminary and final results. First, this is a relatively small study (planned enrollment of 100) which may limit our ability to detect small differences between the intervention and control groups. Second, there may be participation bias in our sample as our preliminary data suggest participants to date have been more tech-savvy than national data (ie, it may be that less tech-savvy patients are less inclined to agree to participate). Third, we did not include education or income in our survey instruments so we will not be able to determine to what extent these factors explain the tech-savviness of our participants, although we will be able to extract other traditional determinates of low technology use such as race/ethnicity and payer status (eg, Medicaid vs Medicare or private insurance) from our EMR for final analysis. Finally, we did not conduct postdischarge interviews so we do not know how patient perspectives on using their PHR might change over time; this is an important question for future studies. Future studies may also consider following patients for longer than 7 days after discharge (eg, 30 days), although we did not find any significant results at 7 days so stronger postdischarge reinforcement may be needed for future hospital-based interventions to create lasting impact.

### Conclusions

We have designed a protocol to examine whether the provision of tablet computers and varying levels of PHR assistance (log-in only for control, extensive guidance for intervention) can improve inpatient use of their PHR to engage in hospital care. Our preliminary results suggest that learning about the device is not a significant barrier and that all patients actively enrolled (intervention and control) use their PHR at rates similar to a passively enrolled virtual cohort of patients who were already engaged with their PHR. Our final results will examine whether the intervention patients are different than control patients in terms of inpatient and postdischarge use of their PHR or whether the mere provision of tablets and log-in assistance in the hospital might be enough to explain higher PHR engagement in these patients. Our results are designed to have real-world impact at our medical center and may have important applications for many other medical centers and health systems that are searching for effective implementation strategies to increase patient engagement with PHR systems already in use in their facilities.

## Appendix

Multimedia Appendix 1 Study protocol details.

Multimedia Appendix 2 Debrief interview data collection tool example.

## Abbreviations

EHR: electronic health record
OR: odds ratio
PCR: personal health record
UCSF: University of California, San Francisco
