# Supplementary material for: Using Tablet Computers to Increase Patient Engagement With Electronic Personal Health Records: Protocol For a Prospective, Randomized Interventional Study
Source: JMIR Res Protoc. 2016 Sep 6;5(3):e176. doi: 10.2196/resprot.4672 (PMC5030452; doi:10.2196/resprot.4672)
Supplement: Multimedia Appendix 1 [file resprot_v5i3e176_app1.pdf]

Appendix 1: Inpatient PHR Engagement Study Protocol

Screening

Project Director (PD) will screen the general medicine department's admissions list daily for newly admitted patients via APeX (UCSF's Electronic Medical Record). The PD will review patient's charts and determine eligibility using the follow inclusion criteria

<u>Inclusion Criteria</u>:

- 18 years and older
- English Speaking
- Not admitted to ICU
- Not cognitively impaired (Alert and Oriented to person, place and time)
- Not under contact, droplet, airborne or radiation isolation

Because, cognitive impairment is the only factor that cannot be directly accessed from this list, further exploration of the patient's notes and/or consulting with the patient's bedside nurse or caregiver may be required. Once a study-specific list of eligible patients has been created, eligible patients will be divided among the PD and 2 Research Assistants (RAs). Each RA/PD will be assigned at least 3 patients to enroll depending on inpatient admission census.

Next, individual RA/PDs will print consent packet (Consent form, HIPAA form and Patient Bill of Rights), and will generate and print MyChart activation codes and Study Check Lists per patient (please see Appendix 2 and 3). Each RA/PD will be have 3 regular sized iPad 2 devices (iPad® 16 GB 3rd generation Model A1430) and a personal iPad Mini (iPad ® 16 GB Model A1432) to enter data in real time when interviewing patients. Now RA/PDs are ready to approach and enroll patients.

Enrollment

<u>Enrollment</u>:

RAs will make sure to knock then enter patient's room, address patient by name, acknowledge family (if present), and introduce themselves by name and role. Once RA has the patient's attention, they can introduce the study. Example Conversation:

"Hello Mr. X. My name is Y, a research assistant from the division of hospital medicine. We are conducting a new research study to better engage patients with their personal health records using tablet computers. Is this a good time to talk to you more about our study and see if you are interested in participating?"

If not interested, thank patient for their time and leave the room. If patient is interest in hearing more, go over the consent form, participation responsibilities, HIPAA form and Bill of Rights. Then explain that we will be setting up their MyChart account (if needed) and leaving them with an iPad for a few hours to explore MyChart's features. Should the patient decided to participate, have them sign the consent and HIPAA forms.

Randomization:

Block Randomization:

Blocks of 10 randomized 1s and 2s were generated with a random number generator (http://stattrek.com/statistics/random-number-generator.aspx). 1 designates intervention and 2 is control. These numbers are placed on a Google spreadsheet that can be accessed remotely by all research assistants. Once a patient has consented to the study, the spreadsheet will be used to determine which group the patient will belong to. RAs will go down the list as each patient is enrolled.

Example:

Randomized #	Study Group	RA Initials	Patient Study ID
2	Control	RJ	45
1	Intervention	AN	47
2	Control	AN	48
2	Control	YM	68
1	Intervention		
1	Intervention		
1	Intervention		
2	Control		
2	Control		
1	Intervention		

Device Orientation:

If patient has never used an iPad before, take a moment to show them the home button, keyboard and zoom features. Next, if needed show patient how to use a touch-screen, check email, use safari or chrome browser. If patient does not have an email address, help him/her create one, as this will be necessary in case MyChart has not been previously activated. If they are familiar with an iPad skip directly to Pre-Survey.

Pre-Survey:

Use the study check list to guide patients through the tasks needed to be completed and remind them that the study ID on this form will be needed to link their pre and post survey responses. Have the patient start the Pre-Survey and complete the questions. Once completed, remind the patient to complete the post-survey before RA picks up the device.

MyChart Activation:

After the pre-survey, have the patients log into MyChart with their information using the shortcut to the website. For those participants without a previous MyChart account, use the generated MyChart activation code, their DOB, and SSN to active their account.

MyChart Tutorial:

Once logged into MyChart provide tutorial depending on intervention/control group:

- **Intervention**: Provide detailed MyChart tutorial, including how to navigate:
    - Personal information (address, phone number, email, etc)
    - Medications tab
    - Refill Medications
    - Test Results
    - Appointment Tabs
    - Message center

- **Control**: Do not provide any tutorial, but encourage the patient to take some time to explore MyChart.

Now inform participant they will have the iPad for several hours for them to become more familiar with tablet computers, explore MyChart and their entertainment. Also, remind them to complete the post-survey the device is retrieved. Make sure they are aware that the iPad is NOT for them to keep. Lastly, let them know that upon collection, the RA will ask the patient to demonstrate a few tasks on MyChart and have the patient answer some questions regarding their experience with MyChart.

Post-Survey:
Survey should be completed at patient's leisure before the iPad is collected. If this does not occur, have patient complete it before collection and debriefing conversation.

Device left with patient until collection around 4pm
RA/PDs will continue with enrollment of additional patients as time permits. Complete first portion of the data tool form on REDCap for each patient enrolled. The other half will be complete during the "debriefing" conversation.

Device Collection and Debrief Interview

Revisit patient and make sure participant has completed the post-survey and proceed with debrief interview (Appendix 2). Ensure that have patients demonstrate whether they can perform each task mentioned in the data tool independently (log in and verify personal information, navigate to medications tab, navigate to medications refills, appointments, test results and messages). Do NOT offer any help unless specifically asked for. Select answers accordingly under the topics covered at debrief section. For instance, leave both blank if patient could not perform the task and did not ask for help, or mark "demonstrated at debrief" if patient could not perform, but desired help. Now, complete the open ended questions and using iPad mini enter responses directly onto REDCap. Try to dig below the surface if patient provides a single word answer (ie. Yes/No). Once debrief is completed and entered into REDCap, reset device by erasing all data and then use the iCloud account to reload iPad to original study settings and be ready for the next participant.
