# Supplementary material for: Using Tablet Computers to Increase Patient Engagement With Electronic Personal Health Records: Protocol For a Prospective, Randomized Interventional Study
Source: JMIR Res Protoc. 2016 Sep 6;5(3):e176. doi: 10.2196/resprot.4672 (PMC5030452; doi:10.2196/resprot.4672)
Supplement: Multimedia Appendix 2 [file resprot_v5i3e176_app2.pdf]

Appendix 2: Debrief interview data collection tool example

Part I. For both groups, intervention and control, ask patient to demonstrate the following functions on MyChart without assistance. If patient is unable to perform task, leave blank. If patient is unable and ask for help, demonstrate task again to patient then make "demonstrated at debrief". NOTE: Do not offer to show the patient unless asked specifically.

Tasks	Accomplished independently	Demonstrated at debrief
Login and verify personal information (address, phone number, email, etc.)		
Navigate to Medications tab		
Navigate to Medication Refills page (and verify pre-admission med list, if applicable)		
Navigate to Appointments tab (and verify appointments, if applicable)		
Navigate to Test Results tab (and view specific test results, if applicable)		
• Navigate to Message tab		

Part II. Please ask the follow questions to the patient regarding their experience with the iPad. If answers are very limited, try to ask follow up questions and dig deeper.

1. Who was interviewed regarding iPad experience?
€ Patient
€ Patient & Caregiver
€ Patient & Family Member(s)

2. How would you describe your overall satisfaction with the iPad today?

3. How could we improve your experience with the device?

4. Did you have difficulty seeing/reading the screen, typing, or pushing buttons to do what you wanted to?

5. Please tell me about accessing MyChart: was it useful to you?

6. What were you able to do using the MyChart today?

7. What barriers or challenges did you encounter in using MyChart?
